# Behavioral Perceptions of Oakland University Female College Students towards Human Papillomavirus Vaccination

**DOI:** 10.1371/journal.pone.0155955

**Published:** 2016-05-20

**Authors:** Aishwarya Navalpakam, Mohammed Dany, Inaya Hajj Hussein

**Affiliations:** 1 Oakland University William Beaumont School of Medicine, Rochester, MI, United States of America; 2 College of Medicine, Medical University of South Carolina, Charleston, SC, United States of America; University of Palermo, ITALY

## Abstract

Human Papillomavirus (HPV) vaccination decreases the risk for cervical cancer. However, the uptake of HPV vaccine remains low when compared with other recommended vaccines. This study evaluates the knowledge and attitudes towards HPV infection and vaccination, and the readiness for the uptake of HPV vaccine amongst female students attending Oakland University (OU) in Michigan, United States. This is a cross-sectional study targeting a randomized sample of a 1000 female OU students using an online questionnaire. The data were statistically analyzed using SPSS software. A total of 192 female students, with the mean age of 24 years completed the survey. The majority of participants had previous sexual experience with occasional use of contraceptives (78.1%), were non-smokers (92.7%), and non-alcohol drinkers (54.2%). The participants had a mean knowledge score of 53.0% with a standard error of 2.3% translating to a moderately informed population. The majority agreed that HPV is life threatening (79%), the vaccine prevents cervical cancer (62%), and that side effects would not deter them from vaccination (63%). Although two thirds (67%) believed that, based on sexual practices in the United States, female college students in Michigan have a higher chance of contracting HPV, about 50% did not believe they themselves were at risk. Higher knowledge correlated with increased recommendation for the vaccine (correlation-factor 0.20, *p* = 0.005). Results suggested that the best predictor for improvement of vaccination was the awareness level and health education. This indicates a need for an educational intervention to raise awareness, increase HPV vaccine uptake, and decrease the incidence of cervical cancer.

## Introduction

According to the Centers for Disease Control and Prevention (CDC), about 6 million newly diagnosed cases of HPV infections are reported every year. In fact, genital infection with HPV is the most common sexually transmitted infection (STI) [1]. HPV types 6 and 11 cause greater than 90% of cases of genital warts and are not carcinogenic [2]. However, HPV types 16 and 18, which cause 10% of genital infections, are responsible for the majority of cases of cervical cancers and neoplasms [2,3]. About 99.7% of cervical cancer cases contain HPV DNA [3]. According to the CDC, every year 12,000 women acquire cervical cancer and 4,000 women die from it in the United States (US). Risk factors for HPV infections include the number of sexual partners, age at first sexual intercourse, sexual behavior of male partners, tobacco smoking, high parity, and long-term use of oral contraceptives [3].

It is well known that, cervical cancer can be prevented if herd immunity and screening are insured. Since the introduction of the Papanicalaou smear in the 1950s, the incidence and mortality from cervical cancer have decreased in the US [4]. In addition, the HPV vaccination, if given to adolescent girls prior to the initiation of sexual activity, can further reduce this burden by preventing HPV infection and cervical cancer [5,6]. Even with the recommendation of its use beginning in June 2006, the vaccination rate is still relatively low when compared with other recommended adolescent vaccines [7]. HPV infection and cervical cancer continue to be a serious public health concern due to the nuances associated with the access to, knowledge about, and attitude towards accepting the vaccine.

The primary objective of this study is to examine the knowledge and attitudes towards HPV infection and vaccination as well as the readiness for the uptake of the HPV vaccine amongst female college students. By exploring the barriers to vaccination, this study aims to develop realistic future educational interventions that would increase vaccination rates and decrease the incidence of the HPV infection and cervical cancer.

## Methods

### Questionnaire

A cross-sectional survey was performed targeting a randomized sample of 1000 female Oakland University students 18 years old and above. The survey instrument was an online self-administered anonymous questionnaire conducted using Google forms (approved questionnaire in S1 Survey file). This questionnaire was adopted from a similar study published by Dany M et.al [8,9]. It was divided into 4 main parts and contained 38 items in total. The first section explored demographics and non-identifying information such as age, smoking and drinking status, sexual experience, college major, and current vaccination status. The second section contained 16 True or False knowledge questions about HPV infection and vaccination. Participants were then provided with educational information about HPV infection and its vaccine. The third section offered 9 questions with a 5-point Likert scale (Strongly agree–Strongly disagree) on the participants’ attitudes regarding HPV vaccine. The fourth section consisted of a 10-point scale for the willingness to receive the HPV vaccine after receiving the educational information and completing the first three sections of the survey. Google forms automatically populated and saved digital responses to a secure database protecting participant confidentiality throughout the survey process.

### Study Invitation

The randomized population of 1000 OU female undergraduate students above the age of 18 years was chosen by convenience and no power calculations were done. The sample was randomized from a list of enrolled students. Selected individuals received survey invitations through Google forms twice a month, for two months totaling 4 study invitations. The 1000 students were divided into 4 groups: 1 group of 400 and 3 groups of 200. The first group of 400 received their survey invitation in the summer of 2014 and 3 groups of 200 received their invitation during the fall of 2014. The Office of Institutional Research and Assessment at Oakland University (OIRA) provided the email addresses. The study invitation was an email that included an information sheet with the study aims and objectives along with the participant’s right to refuse or terminate their participation at any time and without penalty. By clicking on the link to the survey, the participants consented. The completion of the self-administered questionnaire was voluntary, anonymous, and confidential. The survey was available in English, the predominant language at Oakland University.

### Ethical Considerations

The study design and questionnaire content were approved by Oakland University Institutional Review Board. The participants were informed that their participation is entirely voluntary and that they can omit questions they prefer not to answer. All responses amounting from the study were kept strictly confidential.

## Data Analysis

Data analysis was performed by the biostatistician in the Department of Biomedical Sciences at OUWB School of Medicine, Michigan. Statistical analysis was performed using SPSS Statistics for Windows Version 21.0. (Armonk, NY: IBM Corp).

## Results

### Participants’ Characteristics

Out of the 1000 approached individuals, only 192 participants completed the survey. The participation rate was 19.2%. The average age was 24 years with a standard deviation of 6.9 years and a range of 19 to 54 years. Participants’ demographics are summarized in Table 1. Around 46% of the participants were already vaccinated with the HPV vaccine and the remaining 54% were not. While a majority of participants (95.8%) have heard about the vaccine, interestingly 3.6% of people have not heard of its existence. The majority of the participants had previous sexual experience (78.1%), with a majority of them reporting occasional contraception use, while 22.1% had no previous sexual activity. The great majority were non-smokers (92.7%) and did not drink alcohol (54.2%), and only 26% were pursuing or pursued health related majors.

**Table 1:** Characteristics of the study’s female participants.

		n (%)
**Number of Participants**		192(100%)
**College Level**		
	Undergraduate	153 (80.2%)
	Graduate	38 (19.8%)
**Smoking Status**		
	Smoker	9 (4.7%)
	Non- smoker	178 (92.7%)
**Drinking Status**		
	Drinks alcohol	83 (43.2%)
	Does not drink alcohol	104 (54.2%)
**Perceived Economic Status**		
	High and Middle- High	44 (22.9%)
	Middle	90 (46.9%)
	Middle Low and Low	57 (29.7%)
**Sexual History**		
	No sexual experience	44 (22.9%)
	Sexual experience(s) always without the use of contraception	19 (9.9%)
	Sexual experience(s) always with the use of contraception	46 (24.0%)
	Sexual experience(s) sometimes with the use of contraception	82 (42.7%)
**HPV Vaccination Status**		
	Vaccinated	88 (45.8%)
	Not vaccinated	104 (54.2%)
**Awareness of HPV vaccine**		
	Heard about the vaccine before	184 (95.8%)
	Never heard about the vaccine before	7 (3.6%)

The main sources of information about HPV and the HPV vaccine were personal physicians, followed by the media, then family and friends. Educational lectures, the Internet, and other resources were conceived as minor sources of information.

### Knowledge of HPV Infection and Vaccination

The majority of participants (73%) confirmed that HPV infections cause cervical cancer, while a minority (19%) thought that it causes ovarian cancer and the rest (8%) did not know. Although 52% knew that HPV is an STI that can cause genital warts, 73% of the participants thought that only women can be infected with HPV and manifest the symptoms. About two thirds of participants (65%) believed that all HPV infections are caused by one strain of the virus. In addition, 75% of the participants incorrectly answered that the HPV vaccine can only be acquired after the age of 18 years. Moreover, 71% believed that women can only contract the virus from a symptomatic sexual partner and 43% incorrectly believed that HPV infections lead to genital herpes. The rest of the results are exhibited in Table 2.

**Table 2:** Participants’ knowledge of HPV vaccine and infection.

Knowledge Statement	Correct Answer	True n (%)	False n (%)	Do not know n (%)
**Human Papilloma Virus can cause herpes.**	**False**	31 (16.1%)	82 (42.7%)	79 (41.1%)
**Human Papilloma virus can lead to genital warts (growths on the skin of the genitals).**	**True**	99 (51.6%)	32(16.7%)	61 (31.8%)
**In most cases, HPV infected women do not show symptoms.**	**True**	144 (75.0%)	13 (3.8%)	34 (17.7%)
**All HPV infections are caused by the same type of virus.**	**False**	13 (6.8%)	125 (65.1%)	13 (6.8%)
**HPV positive pregnant women can pass the virus to their babies.**	**False**	105 (54.7%)	23 (12.0%)	62 (32.3%)
**Only females can be infected with HPV and show symptoms.**	**False**	17 (18.9%)	141 (73.4%)	34 (17.7%)
**HPV can be transmitted from a carrier to his/her partner only if the carrier shows symptoms.**	**False**	18 (9.4%)	137 (71.4%)	37 (19.3%)
**A normal Pap smear implies that the woman is free of HPV.**	**False**	73 (38.0%)	58(30.2%)	61 (31.8%)
**There is no current cure or therapy for HPV infection.**	**True**	79 (41.1%)	46 (24.0%)	67 (34.9%)
**HPV vaccines have the same effect whether the female takes it before or after being infected with HPV.**	**False**	15 (7.8%)	110 (57.3%)	67(34.9%)
**HPV vaccine can only be taken after the age of 18 years.**	**False**	13 (6.8%)	144 (75.0%)	35 (18.2%)
**HPV vaccination is taken as three injections over a period of six months.**	**True**	136 (70.8%)	16 (8.3%)	40 (20.8%)
**HPV vaccination costs around 30 dollars.**	**False**	40 (20.8%)	16 (8.3%)	136 (70.8%)

After calculations, the combined mean knowledge score was 53.0% with a narrow standard error of 2.3%. This translates that on the average each participant answered half of the knowledge questions correctly. Stratified by different demographic variables, those that were in a health related major had a significantly higher knowledge of HPV (61% ± 19%) than those that were in a non-health related major (50% ± 23%) (P-value = 0.002). The average knowledge score of the undergraduates was 52% ± 23% while the average of the graduates was 58%±18%; however, this difference in means was not significant (p-value = 0.18). In addition, the difference between the other variables such as smoking status, alcohol consumption, vaccination status, and whether the participant heard of HPV as not significant (p-values>0.05), see Table 2.

### Attitudes towards HPV Vaccination

Two thirds (67%) of the participants believed that, based on the general sexual practices in the US, female college students in Michigan have a high chance of contracting an HPV infection; however, about 50% of the participants did not believe that they themselves were at a risk of contracting HPV. In addition, 67% of the participants considered recommending the vaccine to fellow female colleagues and 76% agreed that gynecologists and personal physicians should offer the vaccine to their patients. A majority of the participants agreed that the HPV infection is a serious and life threatening (79%), the vaccine prevents cervical cancer (62%), and that side effects from the vaccine would not deter them from vaccination (63%). However, only 39% considered the vaccine affordable and 33% considered the vaccine as a product produced by pharmaceuticals for non-lucrative reasons. All the participants’ responses to attitude statements are summarized in Table 3.

**Table 3:** Participants’ attitudes towards HPV vaccination statements.

Attitude Statement	Strongly Disagree	Disagree	Neutral	Agree	Strongly agree
**Based on my lifestyle, I believe that I am susceptible for the HPV infection and must get the vaccine.**	54 (28%)	41 (22%)	40 (21%)	34 (18%)	21 (11%)
**Based on the general sexual practice among females in the United States, I believe that female college students in Michigan have a good chance of contracting HPV**	12 (6%)	14 (7%)	38 (20%)	76 (40%)	52 (27%)
**I believe that contracting HPV virus is serious and life threatening.**	2 (1%)	8 (4%)	31 (16%)	77 (40%)	74 (39%)
**I believe that the current HPV vaccine is capable of preventing the occurrence of cervical cancer.**	7 (4%)	17 (9%)	50 (26%)	82 (43%)	36 (19%)
**I believe that the price of the vaccine is affordable given the benefits it offers.**	17 (9%)	54 (28%)	46 (24%)	44 (23%)	31 (16%)
**I believe that the side effects of the vaccine are reasonable and will not deter me from taking the vaccine.**	14 (7%)	13 (7%)	43 (23%)	80 (42%)	41 (21%)
**I believe that the HPV vaccine is different from other marketed vaccines produced by pharmaceutical companies with prime purpose of accumulating profit.**	20 (11%)	38 (20%)	70 (37%)	43 (23%)	19 (10%)
**I believe that all gynecologists should recommend the vaccine to their patients, whether or not they come from conservative families.**	7 (4%)	10 (5%)	29 (15%)	72 (38%)	72 (38%)
**I would recommend this vaccine for my female college friends whether or not they come from conservative families.**	14 (7%)	12 (6%)	36 (19%)	69 (37%)	57 (30%)

### Intentions of Receiving HPV Vaccination

After completion of the survey and the educational material, the intention of receiving HPV was assessed for each participant on a scale of 1 to 10, with 1 being least likely and 10 being most likely. The mean intention score to take the HPV vaccine after fulfilling the survey was 6.51 with a standard deviation of 3.51 and a median of 7.00. Participants who were vaccinated already had a significantly higher average intention score (8.6) of recommending a wide coverage of HPV and more college students should take the vaccine when compared with non-vaccinated participants who had a lower average intention score (4.4) (p˂0.0001) to do so.

### Factors Affecting Knowledge and Attitude

Pearson Correlations relating knowledge and attitudes about HPV were calculated. Participants with higher knowledge about HPV considered recommending HPV vaccines to female college students, with a correlation factor was 0.20 (p = 0.005). In addition, participants with higher HPV knowledge significantly considered that gynecologists should recommend the vaccine to female patients. They also believed that HPV is a serious infection and they would recommend the vaccine to their friends. Moreover, they agreed that the side effects are reasonable and are, therefore, more willing to receive the vaccine. The results of these associations are highlighted in Table 4.

**Table 4:** Correlations between knowledge of HPV (base on computed score) and HPV Opinions.

Opinion	N	Pearson Correlation	P-value
**Based on the general sexual practice among females in the United States, I believe that female college students in Michigan have a good chance of contracting HPV and therefore all college female students should receive the HPV vaccine.**	192	0.20387	0.0046
**I believe that the HPV vaccine is different from other marketed vaccines produced by pharmaceutical companies with prime purpose of accumulating profit.**	190	0.00551	0.9399
**I would recommend this vaccine for my female college friends whether or not they come from conservative families.**	188	0.18974	0.0091
**I believe that all gynecologists should recommend the vaccine to their patients, whether or not they come from conservative families.**	190	0.21967	0.0023
**I believe that the current HPV vaccine is capable of preventing the occurrence of cervical cancer.**	192	0.08440	0.2445
**I believe that the price of the vaccine is affordable given the benefits it offers.**	192	0.01323	0.8555
**I believe that contracting HPV virus is serious and life threatening.**	192	0.14127	0.0506
**I believe that the side effects of the vaccine are reasonable and will not deter me from taking the vaccine.**	191	0.13650	0.0597
**Based on my lifestyle, I believe that I am susceptible for the HPV infection and must get the vaccine.**	190	0.16458	0.0233

A multivariable analysis was conducted in this study assessing for knowledge and attitude. The results are highlighted in Table 5. Knowledge had a beta coefficient of 41.9 for awareness of HPV (p˂0.0001). Greater awareness of HPV correlates with greater knowledge. These results substantiate the need for increasing awareness to increase vaccination rates among college students. In addition, the attitude had a beta coefficient of 0.49 (positive attitude) for vaccination status (p˂0.0001). A more positive attitude towards the vaccine correlates with a higher vaccination status and more likelihood for recommendation to others. This indicates the possible combined impact of increasing awareness leading to increasing vaccination, and increasing recommendation.

**Table 5:** Multivariable analysis for predicting knowledge and attitude scores.

		Beta coefficient	p-value
**Knowledge Score**			
	Age	8.105	0.0161
	Major	8.098	0.0295
	Perceived Economic Status	3.297	0.0770
	Sexual History	2.140	0.0901
	Awareness of HPV	41.876	<0.0001
**Attitude score**			
	Sexual History	0.1141	0.0481
	Vaccination Status	0.4856	<0.0001

## Discussion

While previous studies in this field examined the medical efficacy of the HPV vaccine in preventing cervical cancer, this study explored the HPV infection and vaccination from a social and public health perspective. To achieve complete prevention of any preventable disease, public awareness and health literacy are essential. Even though HPV infections are the most common STIs [1], this study revealed that the participants’ average knowledge of HPV was moderate (53%). This is in strike contrast to knowledge and attitude of college students in the US regarding the Human Immunodeficiency Virus (HIV), which is more sensationalized and feared. In fact, one study reported that around 77% of college students at Midwestern University in the US were familiar with the virus, its mode of transmission, and preventive measures [10]. However, it can be noted that participants with a health- related background were more knowledgeable, more likely to tolerate the vaccine, and more likely to have been already vaccinated.

A national survey analysis revealed that the two most common reasons for parents refusing vaccination for their children were perceived low risk for HPV and institutional barriers [11]. Interestingly, the participants, the adolescents, reflected this denial as well. Many participants considered that based on their sexual practices, they themselves are not susceptible to the infection. However, they believed that their colleagues, in general, are more likely to contract the infection. Although they grasp the need for the vaccine, they perceive themselves as not a part of the issue. This allowed for refusal of the vaccine and a negative attitude towards it.

The major source of information about HPV, in this study, was personal physicians and gynecologists (71%). However, a recent systematic review of the literature showed that there is low knowledge about HPV infection and its relationship with non-cervical cancers/genital warts among health care professionals (HCP) [11]. The HCP’s tended to offer mostly risk-based recommendations on perceived likelihood of the patient acquiring the infection [12]. They also site financial concerns, such as lack of insurance coverage and reimbursement, as barriers to vaccination by HCP’s [12]. Interestingly, parents indicated that receiving a physician recommendation for vaccination is an important factor for accepting vaccination [12]. Therefore, it would be of clinical relevance to assess the contribution of HCP’s to HPV awareness in future studies. Data also revealed that the knowledge about HPV and the vaccine seemed to be more restricted to the health and medical fields, which necessitates a need for health education to be more accessible to the general public without funneling through the barriers faced by HCP’s to providing this information.

It is important to explore the economic burden of HPV and cervical cancer. In fact, cervical cancer disproportionately affects women of socio-economically disadvantaged and ethnic minorities in the United States [13]. Those that face the greatest focus of this disease are most likely to find hurdles to overcome their illness including barriers to health care, lack of social support, and increased stigma associated with acquiring an STI. Furthermore, according to the American Cancer Society, each vaccination costs about $140 and, therefore, the cost of the series could be totaled up to $500. Insurance plans do cover the vaccine; however, teenagers and children under the age of 19, who do not have insurance or are eligible for Medicaid, are covered under Vaccines for Children (VFC) program. Although the cost of vaccination seems exorbitant, screening for and treating cervical cancer, in total, costs up to 6 billion dollars annually in the United States [14]. This makes HPV one of the most expensive sexually transmitted infections to care for, second only to HIV. These expenses and financial barriers can be drastically lowered if more awareness and higher vaccination rates were achieved.

It is encouraging to note that 46% of participants were already vaccinated before completion of the survey, which is significantly higher than similar studies conducted in Lebanon where 16.7% of participants were vaccinated ^8^ and in New Zealand were only 7% were vaccinated [15]. Furthermore, 96% of participants have previously heard of HPV previously and 4% never heard of the virus before the survey. These numbers are significantly higher than those of similar studies conducted in Europe, Asia and Africa whereby only 30% to 40% of participants have heard of HPV prior to the survey [16–18]. Moreover, a positive intention regarding getting the vaccine was inferred after gathering more information and completing the survey. The population in this study was aware of the existence of the virus and further health education could have a positive impact on vaccination rates.

## Limitations

The study sample was chosen by convenience in this study and the targeted population was only female college students attending Oakland University in Rochester, Michigan. Therefore, generalizations from this study cannot be made, as this sample is not representative of the general population. A larger nationwide survey might offer more significant results. This study also focused only on female students and currently the vaccination is recommended for males as well. In brief, a more inclusive survey would offer more impactful information on the barriers to vaccination.

## Conclusion

This study tackled, in depth the knowledge, attitude, and intentions of female university students regarding HPV infections and vaccines in the aim of identifying areas and predictors of improvements. The knowledge about HPV was moderate, and the best predictor of improvement was the awareness and health education levels. Therefore, educational resources and activities, as well as awareness campaigns seem to be an inexpensive and effective way to improve disease knowledge, tolerance, and increase HPV vaccination rates. It would also be relevant to assess the current contribution of HCP and health care systems in the fight against HPV, assess knowledge and attitudes of adolescent males, and look for further sources of improvement.

## Supporting Information

S1 Survey (DOCX)

## References

[1] Gloria YF, Bierman R, Beardsley L, Chang CJ, Burk RD. Natural History of Cervicovaginal Papillomavirus Infection in Young Women. N Engl J Med. 1998;338;7: 423–428. 9459645 10.1056/NEJM199802123380703

[2] Siddiqui M, Asif A, Perry CM. Human Papillomairus Quadrivalent (types 6, 11, 16, 18) Recombinant Vaccine (Gardasil). Adis Drug Profile. 2006;66;9: 1263–1271. 10.2165/00003495-200666090-00008 16827602

[3] Franco EL, Duarte-Franco E, Ferenczy a. Cervical cancer: epidemiology, prevention and the role of human papillomavirus infection. CMAJ. 2001;164;7: 1017–25. 11314432 PMC80931

[4] Benard VB, Thomas CC, King J, Massetti GM, Doria-Rose VP, Saraiya M. Vital signs: cervical cancer incidence, mortality, and screening—United States, 2007–2012. MMWR Morb Mortal Wkly Rep. 2014;63;44: 1004–9. 25375072 PMC5779486

[5] Villa LL, Perez G, Kjaer SK, Paavonen J, Lehtinen M, Muñoz N. Quadrivalent Vaccine against Human Papillomavirus to Prevent High-Grade Cervical Lesions. N Engl J Med. 2007;356;19: 1915–1927. 17494925 10.1056/NEJMoa061741

[6] Garland SM, Hernandez-Avila M, Wheeler CM, Perez G, Harper DM, Leodolter S, et al Quadrivalent vaccine against human papillomavirus to prevent anogenital diseases. N Engl J Med. 2007;356;19: 1928–43. 17494926 10.1056/NEJMoa061760

[7] Bartlett JA, Peterson JA. The uptake of Human Papillomavirus (HPV) vaccine among adolescent females in the United States: a review of the literature. J Sch Nurs. 2011;27;6: 434–46. 10.1177/1059840511415861 21750234

[8] Dany M, Chidiac A, Nassar AH. Human Papilloma Virus vaccination: assessing knowledge, attitudes, and intentions of college female students in a developing country. Vaccine. 2015, 2 18;33;8: 1001–7. 10.1016/j.vaccine.2015.01.009 25597945

[9] Alaaeddine G, Al kuhaimi T, Al assaad R, et al Assessing knowledge and attitudes of owners or managers of hospitality venues regarding a policy banning indoor smoking. Public Health. 2013;127;5: 461–6. 10.1016/j.puhe.2013.01.015 23608025

[10] Inungu J, Mumford V, Younis M, Langford M. HIV Knowledge attitudes and practices among college students in the United States. J Health Hum Serv Adm. 2009 Winter; 32;3: 259–77. 20099580

[11] Liddon NC, Hood JE, Leichliter JS. Intent to receive HPV vaccine and reasons for not vaccinating among unvaccinated adolescent and young women: findings from the 2006–2008 National Survey of Family Growth. Vaccine. 2012;30;16: 2676–82. 10.1016/j.vaccine.2012.02.007 22342548

[12] Holman DM, Benard V, Roland KB, Watson M, Liddon N, Stokley S. Barriers to human papillomavirus vaccination among US adolescents: a systematic review of the literature. JAMA Pediatr. 2014;168;1: 76–82. 10.1001/jamapediatrics.2013.2752 24276343 PMC4538997

[13] Ashing-giwa KT, Kagawa-singer M, Padilla GV, Tejero JS, Chhabra R, Martinez L, Tucker MB. The impact of cervical cancer and dysplasia: a qualitative, multiethnic study. Psychooncology. 2004;13;10: 709–28. 15386644 10.1002/pon.785 PMC1704077

[14] Frazer IH, Cox JT, Mayeaux EJ, Franco EL, Moscicki AB, Palefsky JM, Ferris DG, Ferenczy AS, Villa LL. Advances in prevention of cervical cancer and other human papillomavirus-related diseases. Pediatr Infect Dis J. 2006;25;2 Suppl: S65–81. 16462611 10.1097/01.inf.0000196485.86376.46

[15] Chelimo C, Wouldes T. Human papillomavirus knowledge and awareness among undergraduates in healthcare training in New Zealand. NZMJ. 2009;22: 33–45. 19859090

[16] Francis S, Nelson J, Liverpool J, Soogun S, Mofammere N, Thorpe RJ Jr. Examining attitudes and knowledge about HPV and cervical cancer risk among female clinical attendees in Johannesburg, South Africa. Vaccine. 2010;28: 8026–32. 10.1016/j.vaccine.2010.08.090 20887829

[17] Marek E, Dergez T, Rebek-Nagy G, Krickskovics A, Kovacs K, Bozsa S, et al Adolescents’ awareness of HPV infections and attitudes towards HPV vaccination 3 years following the introduction of the HPV vaccine in Hungary. Vaccine. 2011;29: 8591–98. 10.1016/j.vaccine.2011.09.018 21939711

[18] Kang H, Kim J. Knowledge, Attitudes of human papillomavirus vaccine, and intention to obtain vaccine among Korean female undergraduate students. Women&Health. 2012;51: 759–76. 10.1080/03630242.2011.627091 22185290

