# Supplementary material for: Behavioral Perceptions of Oakland University Female College Students towards Human Papillomavirus Vaccination
Source: PLoS One. 2016 May 20;11(5):e0155955. doi: 10.1371/journal.pone.0155955 (PMC4874592; doi:10.1371/journal.pone.0155955)
Supplement: S1 Survey — (DOCX) [file pone.0155955.s001.docx]

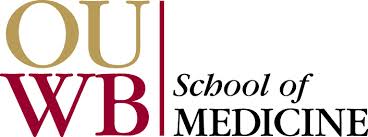


| **Section 1: General Information**  **Kindly answer the following personal non-identifying questions** | | | | | | | | | | | | | | | | | | | | | | | | | |
| --- | --- | --- | --- | --- | --- | --- | --- | --- | --- | --- | --- | --- | --- | --- | --- | --- | --- | --- | --- | --- | --- | --- | --- | --- | --- |
| **1** | **Year of Birth** | 19 __ __ | | | | | | | | | | | | | | | | | | | | | | | |
| **2** | **College Year** | Freshman | | | Sophomore | | | | | | | Junior | | | | | Senior (3^rd^ year or above) | | | | | Graduate | | | |
| **3** | **School** | Arts & Sciences | | Business | | | | | | Education & HRD | | | Engineering & Computers | | | | | Health Sciences | | | | | | Other ____________ | |
| **4** | **Religion** | Christianity | | | | | Judaism | | | | | | | | Islam | | | | | | Other _____________ | | | | |
| **5** | **Perceived Economic Status** | | High | | | | | Middle-High | | | | | | Middle | | | | | Middle-Low | | | | Low | | |
| **6** | **Smoking Status** | | Smoker | | | | | | | | | | | | | Non- Smoker | | | | | | | | | |
| **7** | **Alcohol Drinking Status** | | Drinker | | | | | | | | | | | | | Non-Drinker | | | | | | | | | |
| **8** | **Sexual History** | | No sexual experience  Sexual experience(s) always without the use of contraception (e.g. condom)  Sexual experience(s) always with the use of contraception (e.g. condom)  Sexual experience(s) sometimes with the use of contraception (e.g. condom) | | | | | | | | | | | | | | | | | | | | | | |
| **9** | **Have you been vaccinated before with Human Papilloma Virus (HPV) vaccine?** | | Yes | | | | | | | | | | | | | No | | | | | | | | | |
| **10** | **On a scale of 1-10,: 1 being least likely and 10 being most likely how much are you planning of obtaining the Human Papilloma Virus Vaccine** | | 1 | | | 2 | | | 3 | | 4 | | | 5 | | 6 | | | 7 | 8 | | | 9 | | 10 |
| **11** | **Before this study, have you heard about Human Papilloma Virus Vaccine?**  **(if no skip to section 2)** | | Yes | | | | | | | | | | | | | No | | | | | | | | | |
| **12** | **From where did you hear about the vaccine? (Check all that apply)** | | Media  Internet  Personal physician or Gynecologist  University lectures or Professors  Family or Friends  Others, please specify ____________________ | | | | | | | | | | | | | | | | | | | | | | |

**You need to know this information about HPV and its vaccine before you proceed:**

**Human Papilloma Viruses are a group of human viruses that are capable of causing several types of infections and diseases. They are mainly transmitted through sexual intercourse. Among these are the High Risk HPV viruses (mainly HPV 16 and 18) whose infection is highly correlated with uterine and cervical cancer, a very serious type of cancer.**

**Two HPV vaccines have been designed to reduce the risk of the HPV-based sexually transmitted infections: Gardasil and Cervarix. Females 11 years old and above are advised to take the vaccine.**

**The vaccine consists of three injections over a period of six months. Each injection costs around 130 dollars summing up to a total of 390 dollars for the whole vaccine.**

**The vaccine is more effective as a preventive measure before getting the viral infection as opposed to receiving the vaccine after contracting the virus.**

**Common side effects of the vaccine include: pain and swelling at the injected site, headaches, muscle pain, fatigue, nausea, vomiting, diarrhea, fever, and abdominal pain.**

**Uncommon side effects include upper respiratory tract infection and dizziness.**

| **Section 2: Knowledge about Human Papilloma Virus and its vaccine**  **This section is designed to test your knowledge about Human Papilloma Virus and its vaccine.**  **For each of the following statements, choose the choice you are aware of. If you do not know the answer, just choose the “Do not know” option.** | | | | | |
| --- | --- | --- | --- | --- | --- |
| **The type of cancer highly associated with HPV infection is:** | Ovarian cancer | Breast cancer | Uterine cervical cancer | | Do not know |
| **Human Papilloma Virus can cause herpes** | True | False | | Do not know | |
| **Human Papilloma virus can lead to genital warts (growths on the skin of the genitals)** | True | False | | Do not know | |
| **HPV can be transmitted through vaginal, anal, and oral sex as well as genital to genital contact** | True | False | | Do not know | |
| **In most cases, HPV infected women do not show symptoms** | True | False | | Do not know | |
| **All HPV infections are caused by the same type of virus** | True | False | | Do not know | |
| **HPV positive pregnant women can pass the virus to their babies** | True | False | | Do not know | |
| **Only females can be infected with HPV and show symptoms** | True | False | | Do not know | |
| **HPV can be transmitted from a carrier to his/her partner only if the carrier shows symptoms** | True | False | | Do not know | |
| **A normal Pap smear implies that the woman is free of HPV** | True | False | | Do not know | |
| **There is no current cure or therapy for HPV infection** | True | False | | Do not know | |
| **HPV vaccines have the same effect whether the female takes it before or after being infected with HPV** | True | False | | Do not know | |
| **HPV vaccine is best taken before starting to have sexual activities** | True | False | | Do not know | |
| **HPV vaccine can only be taken after the age of 18 years** | True | False | | Do not know | |
| **HPV vaccination is taken as three injections over a period of six months** | True | False | | Do not know | |
| **HPV vaccination costs around 30 dollars** | True | False | | Do not know | |

|  | | | | | | |
| --- | --- | --- | --- | --- | --- | --- |
| **Section 3: Attitudes towards Human Papilloma Virus vaccination**  **This section is designed to assess your attitudes towards the HPV vaccine after you have gained some general knowledge on the subject.**  **Choose the choice which reflects your opinion of each of the following statements.** | | | | | | |
| **Based on my lifestyle, I believe that I am susceptible for the HPV infection and must get the vaccine.** | Strongly agree | Agree | Neutral | Disagree | Strongly disagree |  |
| **Based on the general sexual practice among females in the United States, I believe that female college students in Michigan have a good chance of contracting HPV and therefore all college female students should receive the HPV vaccine.** | Strongly agree | Agree | Neutral | Disagree | Strongly disagree |  |
| **I believe that contracting HPV virus is serious and life threatening.** | Strongly agree | Agree | Neutral | Disagree | Strongly disagree |  |
| **I believe that the current HPV vaccine is capable of preventing the occurrence of cervical cancer.** | Strongly agree | Agree | Neutral | Disagree | Strongly disagree |  |
| **I believe that the price of the vaccine is affordable given the benefits it offers.** | Strongly agree | Agree | Neutral | Disagree | Strongly disagree |  |
| **I believe that the side effects of the vaccine are reasonable and will not deter me from taking the vaccine.** | Strongly agree | Agree | Neutral | Disagree | Strongly disagree |  |
| **I believe that the HPV vaccine is different from other marketed vaccines produced by pharmaceutical companies with prime purpose of accumulating profit.** | Strongly agree | Agree | Neutral | Disagree | Strongly disagree |  |
| **I believe that all gynecologists should recommend the vaccine to their patients, whether or not they come from conservative families.** | Strongly agree | Agree | Neutral | Disagree | Strongly disagree |  |
| **I would recommend this vaccine for my female college friends whether or not they come from conservative families.** | Strongly agree | Agree | Neutral | Disagree | Strongly disagree |  |

**Section 4: Attitudes towards the Smoke-free Policy**

| **Section 4:**  **After taking this survey,**  **On a scale of 1-10: 1 being least likely and 10 being most likely, How much are you willing now to get vaccinated with HPV vaccine?** | | | | | | | | | |
| --- | --- | --- | --- | --- | --- | --- | --- | --- | --- |
| 1 | 2 | 3 | 4 | 5 | 6 | 7 | 8 | 9 | 10 |
